# Thermocatalytic Behavior of Manganese (IV) Oxide as Nanoporous Material on the Dissociation of a Gas Mixture Containing Hydrogen Peroxide

**DOI:** 10.3390/nano8040262

**Published:** 2018-04-21

**Authors:** Zaid B. Jildeh, Jan Oberländer, Patrick Kirchner, Patrick H. Wagner, Michael J. Schöning

**Affiliations:** 1 Imagine Engineering GmbH, Kopernikusstr. 13b, 50126 Bergheim, Germany; zaid.jildeh@imagine.de (Z.B.J.); patrick.kirchner@imagine.de (P.K.); 2 Institute of Nano- and Biotechnologies (INB), Aachen University of Applied Sciences, Heinrich-Mussmann-Str. 1, 52428 Jülich, Germany; oberlaender@fh-aachen.de; 3 Soft-Matter Physics and Biophysics Section, KU Leuven, Celestijnenlaan 200 D, 3001 Leuven, Belgium; patrickhermann.wagner@kuleuven.be; 4 Institute of Complex Systems (ICS-8), Research Center Jülich GmbH, 52428 Jülich, Germany

**Keywords:** hydrogen peroxide, manganese (IV) oxide, catalytic decomposition, calorimetric gas sensor, numerical simulation

## Abstract

In this article, we present an overview on the thermocatalytic reaction of hydrogen peroxide (H${}_{2}$O${}_{2}$) gas on a manganese (IV) oxide (MnO${}_{2}$) catalytic structure. The principle of operation and manufacturing techniques are introduced for a calorimetric H${}_{2}$O${}_{2}$ gas sensor based on porous MnO${}_{2}$. Results from surface analyses by X-ray photoelectron spectroscopy (XPS) and scanning electron microscopy (SEM) of the catalytic material provide indication of the H${}_{2}$O${}_{2}$ dissociation reaction schemes. The correlation between theory and the experiments is documented in numerical models of the catalytic reaction. The aim of the numerical models is to provide further information on the reaction kinetics and performance enhancement of the porous MnO${}_{2}$ catalyst.

## 1. Introduction

Manganese (IV) oxide (MnO${}_{2}$) is a tetravalent oxide compound of manganese that is commonly found in nature in the form of pyrolusite mineral [1]. MnO${}_{2}$ is thermally stable up to a temperature of about 500 ${}^{\circ }$C, above which thermal decomposition takes place and MnO${}_{2}$ transforms to the trivalent manganese oxide (Mn_2_O_3_) [1,2]. Due to the high activity and thermal stability of MnO${}_{2}$, catalysts made of the bulk material or from a dispersion are widely used in various industrial sectors such as hydrogenation of ethylene or in dry cell batteries as the primary component of the cathode mixture [1,2]. An extensively studied application of MnO${}_{2}$ is in space programs, where it is used as a catalyst for hydrogen peroxide (H${}_{2}$O${}_{2}$)-based fuels [3,4]. In these fuel cells MnO${}_{2}$ catalysts have a high activity that induces an exothermic decomposition reaction of hydrogen peroxide containing solutions [5,6,7,8] or gases [9,10,11,12].

Hydrogen peroxide (H${}_{2}$O${}_{2}$) is known for its strong and fast acting microbicidal properties due to the formation of various radical species that lead to several types of cell damage [13,14,15,16]. Concentrated H${}_{2}$O${}_{2}$ solutions (≥35% *w*/*w*) are established sterilization agents in the food and pharmaceutical aseptic packaging industries, where a high level of disinfection is required [15,17]. A common sterilization procedure for packaging surfaces prior to filling of perishable products at atmospheric conditions is by applying a high-temperature gas mixture of air and gaseous H${}_{2}$O${}_{2}$ with volumetric concentrations up to 8% *v*/*v* and temperatures up to 300 ${}^{\circ }$C [12,15,18]. To produce the H${}_{2}$O${}_{2}$ containing gas, a certain quantity of aqueous H${}_{2}$O${}_{2}$ solution (35% *w*/*w*) is mixed with sterile air and passed through heated channels of a heat exchanger that is designed as an evaporation unit [19].

H${}_{2}$O${}_{2}$ is known for its unstable nature [3,20] and various stabilizing chemicals are added to prevent uncontrolled decomposition caused by the presence of impurities from the production process [14,21]. These stabilizing agents include phosphates, colloids and chelating agents that bind with the impurities to prevent catalytic decomposition of H${}_{2}$O${}_{2}$ [22]. In accordance to the Food and Drug Administration (FDA) Code of Federal Regulations title 21 (21CFR178.1005), the amount of inorganic impurities including stabilizing agents has to meet the specifications set by the Food Chemicals Codex (FCC). Table 1 indicates the allowed level of inorganic impurities as defined by the FCC [23]. The type and concentration of impurities depend on the H${}_{2}$O${}_{2}$ production process and the same holds for stabilizing agents that are chosen in function of the H${}_{2}$O${}_{2}$ application method (bath, spray or vapor sterilization).

The decomposition reaction of H${}_{2}$O${}_{2}$ is a highly exothermic reaction [4,11]. While the auto-thermal decomposition of H${}_{2}$O${}_{2}$ (described in literature as homogeneous decomposition) dominantly occurs at temperatures above 400 ${}^{\circ }$C [24,25], catalysts can be used to lower the activation energy of the decomposition process [26]. Hence, H${}_{2}$O${}_{2}$ decomposition reaction can occur at any temperature (described in literature as heterogeneous decomposition) and about 105 kJ/mol is released [25,27,28].

During the evaporation process of aqueous H${}_{2}$O${}_{2}$ solution in the evaporation unit and dependent on the surface temperature of the fluid channels comprising the evaporation unit, some of the H${}_{2}$O${}_{2}$ stabilizing agents are deposited on the internal surfaces of the evaporation unit. This results in a depletion of original stabilizing agents’ concentration in the H${}_{2}$O${}_{2}$ gas. Consequently, the quantification of H${}_{2}$O${}_{2}$ volumetric concentration exiting an evaporation unit at high temperatures can be unreliable by using conventional analytical methods. In order to maintain a high quality of the final packaged product, a constant sterility assurance level (SAL) must be maintained [13]. In addition to temperature, process time and surrounding humidity, the concentration of the sterilant gas must be kept constant throughout the decontamination process [21,29]. Here, a continuous quantification of H${}_{2}$O${}_{2}$ gas concentration required for sterilization can be achieved by the development of innovative sensing methods and devices.

Recently, several types of H${}_{2}$O${}_{2}$ sensing principles have been studied and reported in the literature. An example of these sensing principles include nanozyme-based colorimetric sensors based on vanadium dioxide (VO_2_) nanofibers [30] or commercially available test kits such as Quantofix (Macherey-Nagel GmbH & Co., KG, Düren, Germany). Moreover, electrochemical sensors have been developed such as a sensor based on platinum nanoparticles [31]. However, these sensor types detect H${}_{2}$O${}_{2}$ concentrations in solutions.

To monitor gaseous H${}_{2}$O${}_{2}$ concentrations, further electrochemical sensors are commercially available, such as Dräger Polytron^®^ 7000 or Vaisala PEROXCAP^®^ sensor. However, these sensor types measure the H${}_{2}$O${}_{2}$ concentration enriched in an electrolyte, which is not applicable under elevated temperature conditions >70 ${}^{\circ }$C.

**Figure 1:**
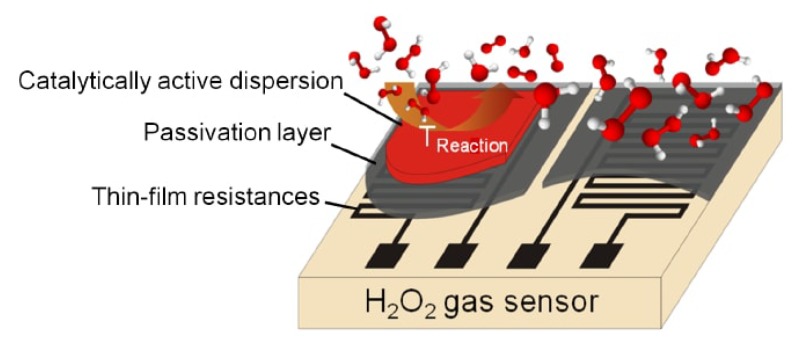
Design of the differential set-up of a calorimetric gas sensor consisting of two chemically passivated thin-film meander sensing elements. One element measures the gas temperature as a reference signal and the other element measures the temperature rise due to an exothermic catalytic decomposition of H${}_{2}$O${}_{2}$. Reproduced with permission from [35]. John Wiley and Sons, Inc., 2012.

Consequently, another sensor type to monitor and determine the concentration of gaseous H${}_{2}$O${}_{2}$ at elevated temperatures as applied during sterilization processes in aseptic filling machines is based on the calorimetric principle. Such calorimetric sensors have been previously developed in our research institute [10,12,18,19,27,32,33,34,35,36]. Hereby, a differential calorimetric sensor set-up was concepted, fabricated and tested to monitor the concentration of H${}_{2}$O${}_{2}$ containing gas at elevated temperatures. The design of the sensor (schematically shown in Figure 1) is based on two equal temperature sensing elements fabricated on a substrate surface. MnO${}_{2}$ powder (Merck KGaA, Darmstadt, Germany) acting as the catalytic material is then deposited on top of one of the sensing elements defining the active sensing element to H${}_{2}$O${}_{2}$, while the other sensing element is specially passivated against H${}_{2}$O${}_{2}$.

At the catalytically activated element H${}_{2}$O${}_{2}$ decomposes to water vapor and oxygen and heat is released in this process. Thereby, the active sensing element records a higher temperature signal than the passive element. By monitoring the temperature difference between the active and passive sensing element, quantification of the released thermal energy on the catalytic surface is possible. The fraction of H${}_{2}$O${}_{2}$ in the gas atmosphere can hence be derived and the concentration logged [26,32]. As a result, calorimetric H${}_{2}$O${}_{2}$ sensors based on MnO${}_{2}$ present a method for the detection, quantification and monitoring of the H${}_{2}$O${}_{2}$-rich environment required in aseptic filling machines.

There is a wide range of catalysts that decompose H${}_{2}$O${}_{2}$. For instance, metallic oxides of lead, silver, cobalt, copper and iron have been reported to decompose H${}_{2}$O${}_{2}$ vapor [9]. Group VIII metals including platinum, palladium and rhodium exhibit exothermic decomposition reactions with H${}_{2}$O${}_{2}$ solutions [37]. Additionally, catalysts of platinum, palladium and silver supported on aluminum oxide are reported to decompose H${}_{2}$O${}_{2}$ vapor [38]. Porous heterogeneous catalytic structures made of oxides of manganese (Mn${}_{x}$O${}_{y}$) are also commonly researched H${}_{2}$O${}_{2}$ catalysts [5,8,39]. In the last decade, catalytic structures based on MnO${}_{2}$ are tested as a compatible active substance of calorimetric H${}_{2}$O${}_{2}$ gas sensors [12,36,40].

A variety of decomposition reaction schemes between H${}_{2}$O${}_{2}$ and the MnO${}_{2}$ catalyst has been proposed in literature [5,11,20]. Equations (1)–(5) provide the most probable reaction pathway of catalytic H${}_{2}$O${}_{2}$ decomposition [5,9,12,20,38]. After a convective mass transport of chemicals to the catalyst surface, the H${}_{2}$O${}_{2}$ molecule is adsorbed on the surface sites [4,41]. The reaction hence starts with electron exchange (Equation (1)) between the H${}_{2}$O${}_{2}$ molecule and MnO${}_{2}$, where a perhydroxyl (HO_2_^·^) radical is formed in the process [7,14,38]. The next step involves chain propagation, where hydroxyl (HO^·^) radical (Equation (2)) and additional HO_2_^·^ are formed (Equation (3)). As a final step the catalyst is replenished (Equation (4)).

(1) $${\mathrm{Mn}}^{4+}+H_{2}O_{2}\to H^{+}+{\mathrm{Mn}}^{3+}+{\mathrm{HO}}_{2}{}^{\cdot }$$

(2) $${\mathrm{HO}}_{2}{}^{\cdot }+H_{2}O_{2}\to H_{2}O+O_{2}+{\mathrm{HO}}^{\cdot }$$

(3) $${\mathrm{HO}}^{\cdot }+H_{2}O_{2}\to H_{2}O+{\mathrm{HO}}_{2}{}^{\cdot }$$

(4) $${\mathrm{HO}}^{\cdot }+{\mathrm{Mn}}^{3+}\to {\mathrm{Mn}}^{4+}+{\mathrm{OH}}^{-}$$

Furthermore, a water molecule is formed as the product of the reaction between a hydroxide and a hydrogen ion as presented in Equation (5). An alternative reaction pathway involves the reaction between HO^·^ and HO_2_^·^ radicals as shown in Equation (6) [33].

(5) $$H^{+}+{\mathrm{OH}}^{-}\to H_{2}O$$

(6) $${\mathrm{HO}}_{2}{}^{\cdot }+{\mathrm{HO}}^{\cdot }\to H_{2}O+O_{2}$$

The reaction scheme in Equation (1) indicates a change in the oxidation state of the manganese from Mn^4+^ as in MnO${}_{2}$ to Mn^3+^ as in Mn_2_O_3_, which correlates with various observations in literature [8,11,12,39,42]. It is also reported that the change in the oxidation state is accompanied by a decrease of the catalytic activity [8]. This can be explained by examining the activation energy of the decomposition of H${}_{2}$O${}_{2}$ on Mn_2_O_3_, which resides at a value of 77.1 kJ/mol [42]. The activation energy of the decomposition of H${}_{2}$O${}_{2}$ on MnO${}_{2}$ is reported to be around 50 kJ/mol [43]. Additionally, the catalyst replenishment reaction shown in Equation (4) requires an electron donor that comes in the form of an HO^·^ radical. Nonetheless, the radical is also used in other reactions as seen in Equation (3) and Equation (6). Therefore, by favoring Equation (3) over the catalyst replenishment (Equation (4)) a decrease of catalyst activity can be expected. In addition to the decrease of activity, catalyst poisoning might occur due to the presence of stabilizing agents in the sterilant gas that can cover the surface of the catalyst preventing the contact with H${}_{2}$O${}_{2}$ gas [44]. Both factors might affect the sensitivity of the calorimetric sensor to H${}_{2}$O${}_{2}$ gas.

In general, the overall catalytic decomposition reaction of H${}_{2}$O${}_{2}$ on a heterogeneous catalyst is mostly expressed in literature as a single-step reaction by neglecting the intermediate products [3,8,39]. In this context, H${}_{2}$O${}_{2}$ is directly converted to its final products and a first-order reaction takes place as expressed in Equation (7). The global reaction rate constant (*k*) of this reaction is determined by an Arrhenius law (Equation (8)). It is worth mentioning that other reaction schemes for the decomposition of H${}_{2}$O${}_{2}$ can follow another reaction order. For example, Satterfield and Stein [25] derived a reaction order of 3/2 for the homogeneous decomposition of H${}_{2}$O${}_{2}$ by proposing a different reaction steps than presented in Equations (2) and (3). A review by Garwig [3] of the heterogeneous decomposition of H${}_{2}$O${}_{2}$ with different inorganic solid catalysts show that the reaction follows first-order kinetics.

(7) $$H_{2}O_{2}\overset{k}{\to }H_{2}O+\frac{1}{2}O_{2}$$

(8) $$k=A_{0}\cdot \exp\left(\frac{-E_{a}}{RT}\right)$$

In Equation (8), $A_{0}$ is the pre-exponential or frequency factor (usually described in 1/s), $E_{a}$ is the activation energy of the reaction in J/(kg·K), *R* is the universal gas constant (8.314 J/(mol·K)) and *T* is the temperature of the reactant at the Kelvin scale. Table 2 introduces the parameters ($A_{0}$ and $E_{a}$) that describe the reaction kinetics as reported in prior literature articles. Using the noted reaction parameters, the *k* value was derived for the decomposition reaction of H${}_{2}$O${}_{2}$ containing gas at a temperature of 270 ${}^{\circ }$C (543.15 K).

Additionally, the $A_{0}$, $E_{a}$ and *k* values are reported in literature to relate to the state of H${}_{2}$O${}_{2}$ (liquid or gas) during decomposition and to the dimension and material of the catalyst [39,49]. The large deviation in the pre-exponential factor leads to a deviation in the rate of catalytic decomposition reaction. Therefore, the nature of the catalytic material and its surface including the distribution and size of the nano-porous particles of the MnO${}_{2}$ play a role in the rate kinetics.

In this work, an analysis of a calorimetric H${}_{2}$O${}_{2}$ sensor and its MnO${}_{2}$-catalyst structure will be presented. Also the surface characterization of the employed MnO${}_{2}$ catalyst by X-ray photoelectron spectroscopy (XPS) and by scanning electron microscopy (SEM) will be addressed. Also, changes in the MnO${}_{2}$ catalyst due to H${}_{2}$O${}_{2}$ decomposition are noted and discussed. To explain the operational principle of the calorimetric H${}_{2}$O${}_{2}$ gas sensor, an exemplary response curve is introduced and analyzed. In the sequel of this work, numerical modeling based on finite-element-method (FEM) combined with SEM images is applied to derive characteristic properties of the porous MnO${}_{2}$-catalyst structure. This is achieved by analyzing the flow through the catalytic structure at the micro-scale to derive the porosity and permeability values of the porous material. Finally, the decomposition reaction is then numerically analyzed at the macro-scale by using the derived characteristics of the porous layer to provide further insight on the reaction through the MnO${}_{2}$ catalyst.

## 2. Calorimetric H${}_{2}$O${}_{2}$ Sensor

### 2.1. Design

**Figure 2:**
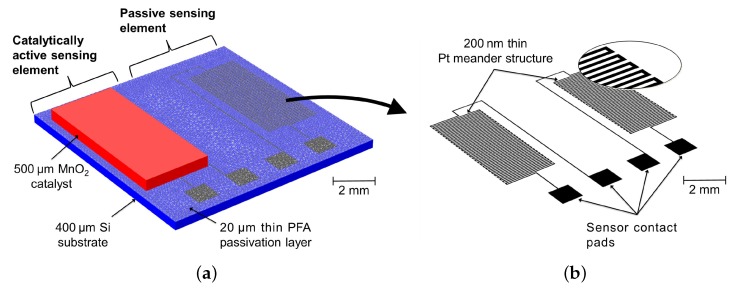
(**a**) Design and dimensions of the developed calorimetric H${}_{2}$O${}_{2}$ sensor based on a Si substrate (blue). The differential set-up consists of sensing elements made of Pt covered with PFA layer. A MnO${}_{2}$-catalyst structure (red) is deposited on top of one sensing element to act as sensing element for H${}_{2}$O${}_{2}$; (**b**) Design of the meander Pt structure (black) that is located underneath the PFA passivation layer. Reproduced with permission from [28]. John Wiley and Sons, Inc., 2017.

The design of the calorimetric H${}_{2}$O${}_{2}$ sensor is shown in Figure 2. For detailed information on the fabrication process we refer to refs. [33,34]. The functional layers of the calorimetric sensor are deposited onto a 400 μm thin silicon (Si) substrate with a 500 nm thin silicon oxide (SiO_2_) layer. Physical vapor deposition is used to deposit a layer of 20 nm titanium (Ti) and 200 nm platinum (Pt). The Ti layer acts as an adhesive between the SiO_2_ and Pt layers. The meander temperature sensing elements are then patterned by a conventional lithography process [33]. Due to the reactivity of H${}_{2}$O${}_{2}$ on Pt [37], the temperature elements are passivated with a 20 μm layer of perfluoralkoxy (PFA) material via spin-coating followed by thermal curing. At this point, the sensor is capable of measuring the temperature of a H${}_{2}$O${}_{2}$-rich environment or flow. To complete the differential design of the calorimetric sensor principle, a dispersion containing MnO${}_{2}$ particles in the range of 10 to 100 μm embedded in an adhesive such as SU-8 (MicroChem Inc., Westborough, MA, USA), is developed on top of one of the passivated Pt sensing elements via drop-coating followed by thermal curing. The final thickness of the catalyst is about 500 μm.

The result of the fabrication process is a differential set-up that represents the calorimetric H${}_{2}$O${}_{2}$ sensor. The passive side of the sensor will measure the temperature of the H${}_{2}$O${}_{2}$-rich gas, while a catalytic exothermic decomposition reaction occurs on the active sensor side releasing heat. The difference in temperature read-out between the passive and the active Pt resistance correlates to the concentration of H${}_{2}$O${}_{2}$ gas in the atmosphere or gas flow.

### 2.2. Sensor Measurements

In the last decade, different configurations of the calorimetric H${}_{2}$O${}_{2}$ gas sensor have been developed and characterized. Näther et al. [19] introduced a differential sensor set-up consisting of two temperature detectors (one catalytically activated and the other one passivated). The work showed a proof-of-concept for a calorimetric detection principle of H${}_{2}$O${}_{2}$ gas in industrial aseptic filling machines. To miniaturize the sensor set-up, monolithic thin-film thermopile sensors based on Si substrate have been developed [32]. To further increase the performance of the sensor, such as the sensitivity toward H${}_{2}$O${}_{2}$ gas, a set of temperature resistors was fabricated on a Si substrate [10]. The corresponding sensor configuration is depicted in Figure 2. With this sensor configuration different types of catalysts (Pd, MnO${}_{2}$ and platinum black (Pt)) as the active sensing element have been studied [10]. In a later work, different polymer-based passivation materials have been characterized [35]. From the published response behavior of these sensors it has been concluded that the sensor with MnO${}_{2}$ catalyst and PFA passivation layer provides the highest sensitivity. To further improve the sensor application and sensitivity the sensor configuration has been fabricated on a flexible polyimide film as the sensor substrate [33,34].

**Figure 3:**
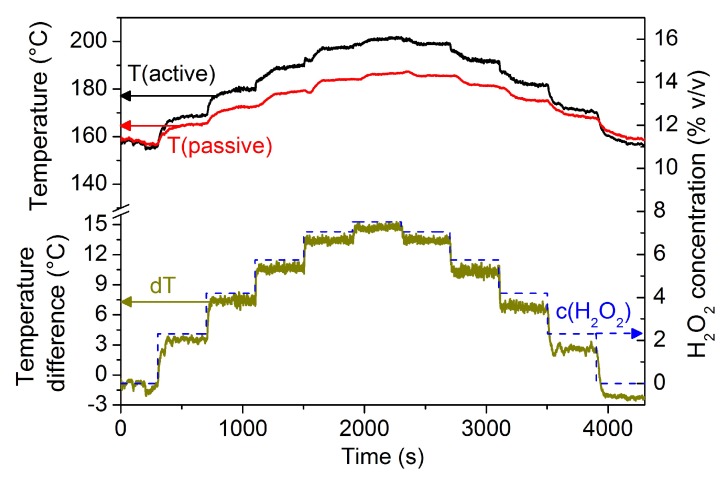
Response graph of a H${}_{2}$O${}_{2}$ gas sensor. The upper part of the graph shows the recorded temperature value from the catalytically active sensing element (black) and the passive sensing element (red). The lower half of the graph shows a step-wise change in the H${}_{2}$O${}_{2}$ concentration (c(H${}_{2}$O${}_{2}$) in dashed blue with the scale to the right) in an outlet stream and the resulting temperature difference (dT in green) as the output signal from the differential sensor set-up (T(active)–T(passive)).

An exemplary response graph of such a calorimetric H${}_{2}$O${}_{2}$ gas sensor is shown in Figure 3. Hereby, a stream containing H${}_{2}$O${}_{2}$ gas is regulated to a predefined temperature and is directed toward the surface of the sensor placed 5 cm below the exit nozzle of the evaporation unit of a sterilization test rig (as described in [19,32]). As explained in Section 2.1, a catalytic exothermic reaction takes place on the MnO${}_{2}$ catalyst which is located on top of one of the sensing elements. The amount of heat released by the exothermic reaction is proportional to the H${}_{2}$O${}_{2}$ gas concentration in the stream or in the surrounding environment. On the one hand, a step-wise change in the recorded temperature signal of the catalytically active sensing element is observed (black curve). On the other hand, the passive sensing element exhibits a similar but lower step-wise change in the recorded temperature signal (red curve). Nonetheless, due to the passivation of the sensing elements by PFA material an exothermic reaction on the passive sensing element does not occur. Therefore, the recorded temperature increase is due to the heating by the gas stream and the conductive heat transfer from the catalytically active sensing element. The temperature difference between the signal of the two sensing elements correlates to the concentration of H${}_{2}$O${}_{2}$ gas. As a result, sensor response graphs similar to the one presented in Figure 3 can be used to obtain a calibration curve and a response function for determining the H${}_{2}$O${}_{2}$ concentration.

### 2.3. Scanning Electron Microscopy (SEM)

**Figure 4:**
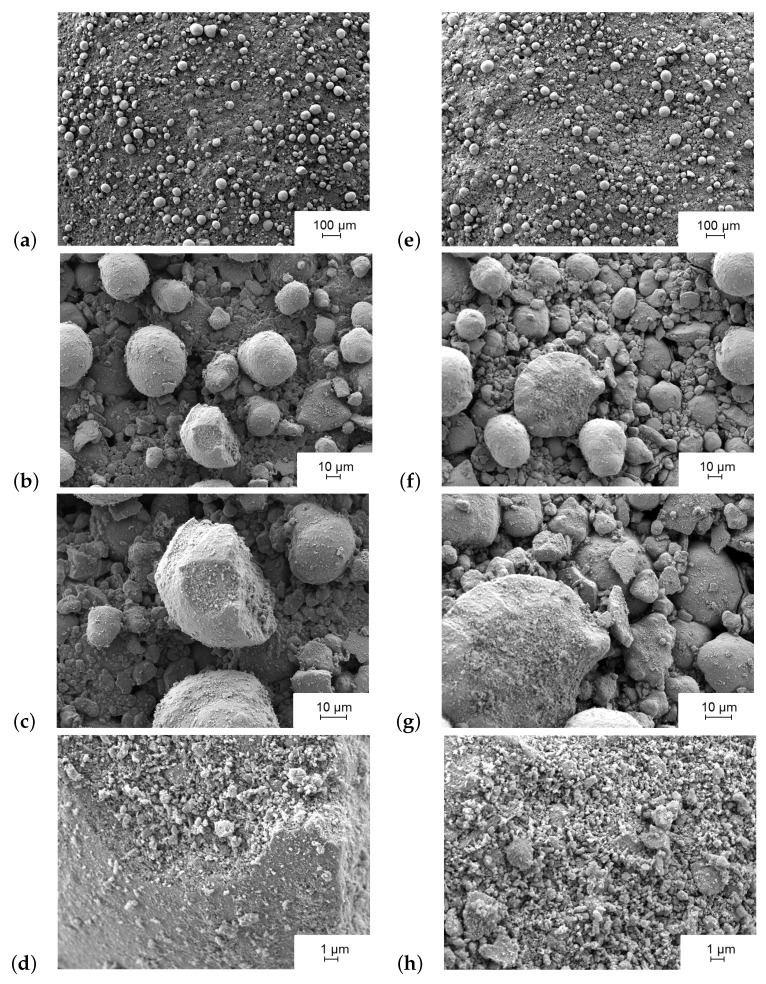
Scanning electron micrographs of MnO${}_{2}$ catalytic layer after fabrication (**a**–**d**) and after surface conditioning with 8% *v*/*v* H${}_{2}$O${}_{2}$ at a temperature of 270 ${}^{\circ }$C (**e**–**h**).

Figure 4 shows the SEM characterization (JSM-7800F, Joel Ltd., Akishima, Tokyo, Japan) of the MnO${}_{2}$ catalytic layer at different magnification levels and in different states. The first state shown in Figure 4a–d represents the catalytic layer after fabrication and prior to the conditioning with H${}_{2}$O${}_{2}$ gas (Solvay, INTEROX^®^ AG Spray 35S). The surface of the catalyst at this point shows the MnO${}_{2}$ particles in bright contrast to the surface. The difference in contrast is related to the surface roughness and hence the rate of electron dispersion. Therein, the catalyst is a mixture of MnO${}_{2}$ particles embedded in an adhesive matrix of SU-8 [12]. Prior to coming in contact with H${}_{2}$O${}_{2}$ in a process called sensor conditioning, the catalyst exhibits a surface that is mostly darker (see Figure 4b) than after H${}_{2}$O${}_{2}$ contact (see Figure 4f). The reason is dominantly due to the adhesive layer that covers the catalyst surface resulted from thermal curing of the MnO${}_{2}$ emulsion. The results of the current SEM characterization match previously published observations presented in ref. [12].

Figure 4e–h depicts the catalytic surface after conditioning with 8% *v*/*v* H${}_{2}$O${}_{2}$ containing gas for more than 4 h at a gas temperature of 270 ${}^{\circ }$C, similar to the procedure explained in ref. [12]. At first glance the particles of MnO${}_{2}$ seem to be sharper defined as compared to the images taken prior to H${}_{2}$O${}_{2}$ conditioning. SEM characterization shows an increase in the surface roughness and the protrusion of MnO${}_{2}$ particles. One possible reason is the removal of the adhesive layer of SU-8 that partially overlaps the MnO${}_{2}$ particles due to the exothermic decomposition of H${}_{2}$O${}_{2}$ on the MnO${}_{2}$ surface. A probable removal mechanism is by the oxidative stress formed from H${}_{2}$O${}_{2}$ vapor, which is comparable to oxygen plasma cleaning as stated in ref. [12]. Additionally, the catalytic decomposition reaction of H${}_{2}$O${}_{2}$ on the sensor surface releases a high level of thermal energy (about 105 kJ/mol), which leads to a localized temperature increase of the MnO${}_{2}$ particle’s surface before dissipating to the rest of the catalyst body and the sensor chip. Hence, we observe that the adhesive SU-8 layer diminishes by the H${}_{2}$O${}_{2}$ decomposition process. This can be due to the thermal stability limit of SU-8 material (about 380 ${}^{\circ }$C [50]) that is reached by exothermic decomposition of H${}_{2}$O${}_{2}$ near the material. Finally, the overall decomposition reaction (Equation (7)) releases gaseous species (water vapor and oxygen) that, if developed in the region between MnO${}_{2}$ particles, the adhesive layer can peel off.

Figure 4d,h present the nanoporous structure of the MnO${}_{2}$ catalyst. The high surface porosity and the scale of porous surface assure a large surface area of the catalyst and explain the high reactivity of the MnO${}_{2}$ catalyst to H${}_{2}$O${}_{2}$ gas compared to other tested materials (see ref. [10]). The effect of H${}_{2}$O${}_{2}$ gas treatment is also notable on the nanoporous structure of MnO${}_{2}$ particle. The surface roughness of a MnO${}_{2}$ particle after peroxide treatment (see Figure 4h) is more pronounced than before the treatment (see Figure 4d).

### 2.4. X-ray Photoelectron Spectroscopy (XPS)

**Figure 5:**
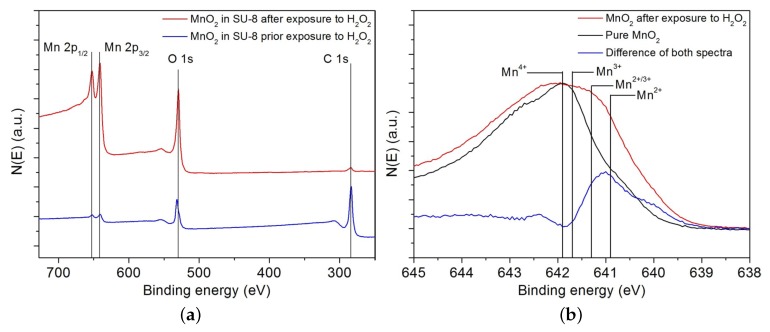
XPS scans of (**a**) dispersion of MnO${}_{2}$ powder in a SU-8 matrix before (bottom curve, blue) and after (top curve, red) exposure to H${}_{2}$O${}_{2}$ vapor and (**b**) magnification of the Mn 2p${}_{3/2}$ peak to assess the state of the MnO${}_{2}$ catalyst before (bottom curve) and after (top curve) exposure to H${}_{2}$O${}_{2}$ vapor. Reproduced with permission from [12]. John Wiley and Sons, Inc., 2014.

The surface characterization of the MnO${}_{2}$ catalyst prior and after the exposure to H${}_{2}$O${}_{2}$ vapor was performed by high resolution XPS analysis using 5600 LS electron spectrometer (Physical Electronics, Inc., Chanhassen, MN, USA) equipped with a small-spot X-ray source providing monochromatic Al Kα photons. Figure 5a represents the first set of experimental data that aim to analyze the effect of H${}_{2}$O${}_{2}$ vapor exposure on the surface of the dispersion containing the MnO${}_{2}$ catalyst. Figure 5b presents the second set of experiments that aim at studying the effect of H${}_{2}$O${}_{2}$ decomposition on the oxidative state of MnO${}_{2}$.

The spectroscopic results in Figure 5a prior and after H${}_{2}$O${}_{2}$ exposure show peaks at binding energy levels of 652.8 eV and 641.2 eV, respectively. These levels can be assigned to the photoelectrons emitted from the Mn 2p core doublet. A peak at the binding energy of 528.8 eV indicates the presence of oxygen atoms. These peaks are stronger after conditioning the catalyst with H${}_{2}$O${}_{2}$ gas. When we take a look at the peak at the binding energy of 287 eV that presents carbon atoms we notice a smaller peak level after conditioning with H${}_{2}$O${}_{2}$ accompanied by an increase in the peak of the binding energy related to the Mn atom. Therefore, the XPS analysis confirms the observation made in Section 2.3 regarding the presence of a thin polymer layer of SU-8 that suppresses the Mn signal of the MnO${}_{2}$ catalyst before the exposition to H${}_{2}$O${}_{2}$ gas.

Figure 5b is a high resolution scan focusing on the Mn 2p${}_{3/2}$ region in order to assess the oxidation state of Mn before and after contact with H${}_{2}$O${}_{2}$ gas. Prior to coming in contact with H${}_{2}$O${}_{2}$ gas, the XPS analysis of the catalyst shows a peak around the binding level of Mn^4+^ that indicates manganese (IV) oxide. After catalyst exposure to H${}_{2}$O${}_{2}$ gas, the peak widens and covers different energy levels. These levels indicate a change in oxidation state of MnO${}_{2}$ to Mn_2_O_3_ [12]. The change in oxidative state confirms the decomposition reaction pathway described by Equation (1).

## 3. Numerical Models

In Section 3.1 numerical modeling is used to analyze the catalytic surface on the micro level. Later on, results from micro-level characterization will be used as an input to evaluate the decomposition reaction of H${}_{2}$O${}_{2}$ containing gas on the macro level (Section 3.2). The term “micro” relates to the catalytic bulk surface and not to the surface of a single MnO${}_{2}$ particle. While the term “macro” represents the catalyst with a thickness of about 500 μm (see Figure 2).

### 3.1. Surface Characteristics of the Nanoporous Catalyst

The sensitivity of the calorimetric sensor to H${}_{2}$O${}_{2}$ gas is affected by different physical parameters related to the nature of the catalyst. The surface porosity (ϵ) and permeability (κ) are two characteristics of a catalyst and its surface. The porosity describes the volumetric or surface ratio of voids in-between the particles and the total volume or surface of catalyst [51]. When the porosity reaches a value of 0, the catalyst is a solid structure, while a porosity value higher than 80% describes a fibrous material [51,52]. The permeability quantifies the resistance of the material for a fluid flow. Hence, an increase in permeability induces higher volumetric flow rates that enhance mass transport of materials and the rate of catalytic reaction. However, the permeability of the MnO${}_{2}$ catalyst is dependent on different factors such as the geometric irregularity of the particles, their size distribution and the arrangement of the channels [53]. This leads to different catalysts with similar porosity value but a different permeability value. For these reasons, direct correlation between the porosity and permeability variables is not straightforward to derive [53].

**Figure 6:**
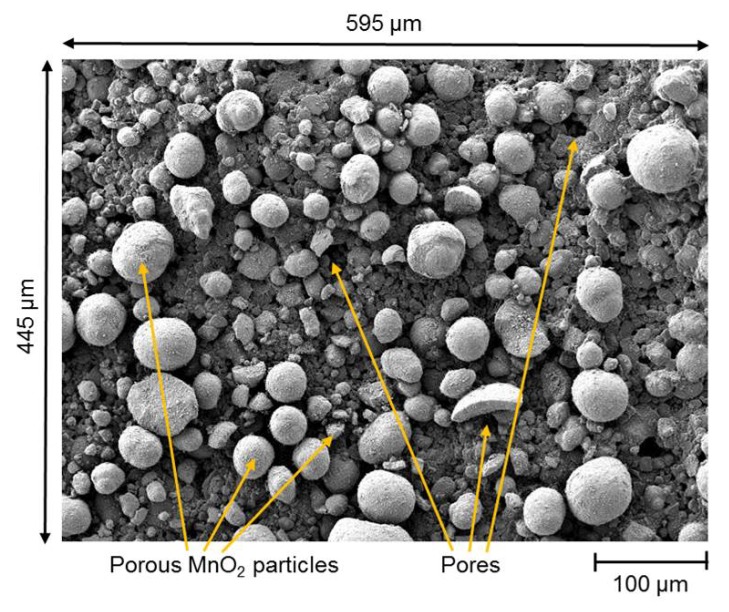
Close-up SEM image of the porous MnO${}_{2}$ catalyst showing MnO${}_{2}$ particles in bright color and the catalyst floor made of the MnO${}_{2}$ dispersion in dark gray.

In this work, numerical modeling of a creeping flow on the surface of the MnO${}_{2}$ catalyst was used to determine the values of ϵ and κ for the catalytic surface. The value of ϵ for the layer is determined as the ratio of the area between the catalyst particles to the total surface of the layer. Figure 6 shows a typical SEM image of the catalytic structure prior to the conditioning with H${}_{2}$O${}_{2}$. The image was chosen due to the clear grey scale contrast between the MnO${}_{2}$ particles and the floor of the chip surface. Here, the SEM image is assumed to be representative for the surface of the functionalized chip. Therefore, to determine the surface area, Figure 6 was imported to a numerical FEM software COMSOL Multiphysics^®^ (Comsol Multiphysics GmbH, Göttingen, Germany).

**Figure 7:**
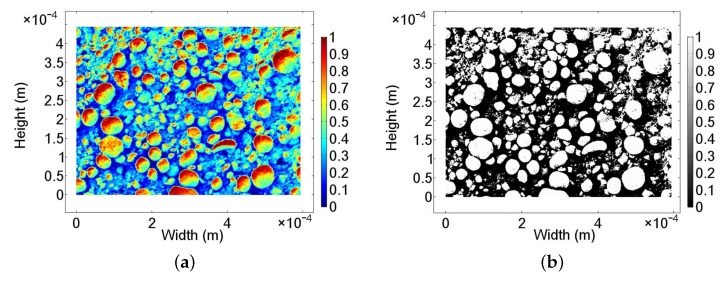
SEM images: (**a**) after importing to the numerical software and defining a color spectrum and (**b**) filtered surface of the MnO${}_{2}$ particle layer.

**Figure 8:**
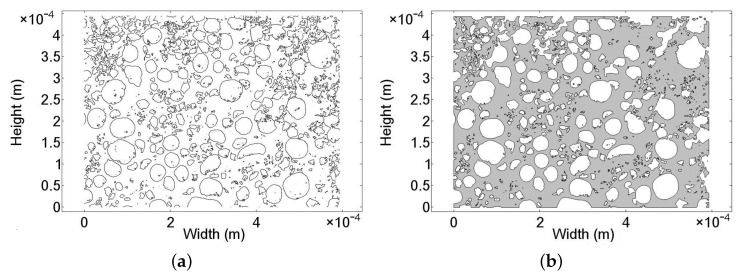
(**a**) Contour of the MnO${}_{2}$ particle and (**b**) numerical domain for flow simulation representing the particle-free region.

A representation of the SEM photo after importing to the numerical model is given in Figure 7a. The color contrast of the SEM image is representative to a normalized height profile of the catalyst. The bright end of the color scale (value > 0.5) shown in Figure 7a indicates MnO${}_{2}$ particles, while the dark regions (value < 0.5) of the spectrum imply their absence and the pore regions. To determine the value of ϵ and κ, the particle-free region is of interest. Hence, the regions containing MnO${}_{2}$ particles are filtered out, resulting in the structure shown in Figure 7b. Subsequently, the contour of the MnO${}_{2}$ particles is defined by the lines at singular value of 0.5. The final particle contours are presented in Figure 8a. The porous flow region is derived from a geometric Boolean operation between the contour lines of the particle and a rectangular domain with identical dimension to the layer. Due to the complexity and the level of geometric details, some geometric parts of the catalytic surface are lost. However, the level of lost details is small in comparison to the overall structural dimensions. As a result, the porous flow region of MnO${}_{2}$ catalyst is approximated numerically in Figure 8b.

The dimensions of the SEM image (Figure 6) are 594 μm in width and 445 μm in height, making a total surface area of 0.26 mm${}^{2}$. The surface integration of Figure 8b produces a value of 0.16 mm${}^{2}$. Thus, the resultant porosity of the surface structure can be calculated as ϵ= 61%.

To derive the permeability of the porous structure, the transversal flow through the catalyst is numerically solved. Darcy’s law (see Equation (9)) is used to resolve the medium flow through a porous material driven by a pressure difference [53,54]. Using numerical methods and Equation (9), the κ value of the catalytic structure can be derived. An empirical form of Darcy’s law is presented in Equation (10).

(9) $$\nabla P+\frac{\mu }{\kappa }U_{D}=0$$

(10) $$U_{i}=\frac{\kappa }{\mu }\frac{\partial P}{\partial x_{i}}$$

Here, $U_{D}$ and $U_{i}$ are the resolved Darcy velocity and its field in m/s, respectively. The parameter μ is the dynamic viscosity of the fluid in Pa·s, P is the pressure value in Pa and $x_{i}$ is the coordinate axes. For a two-dimensional model, ∂P is the pressure difference derived from the boundaries of the model in Pa and $\partial x_{i}$ is the length the flow travels from inlet to outlet boundaries (width of the model) in m. $U_{i}$ represents the overall bulk velocity (Darcy velocity) of the flow in m/s, which is derived by dividing the volumetric flow rate or Darcy flux (resulted from flow simulation) over the inlet surface area of the model.

**Figure 9:**
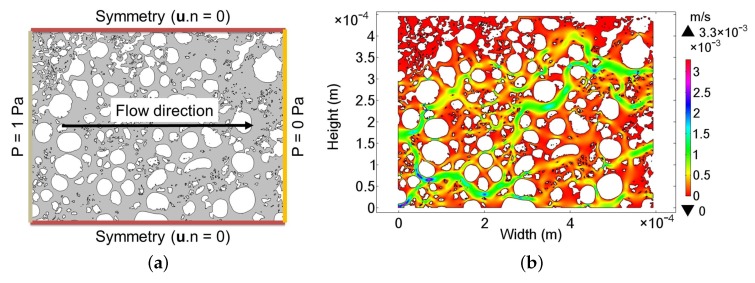
(**a**) Schematic diagram of the numerical simulation design and (**b**) result of an 8% *v*/*v* H${}_{2}$O${}_{2}$ containing gas flow simulation with a pressure difference of 1 Pa.

Equation (9) is valid for low laminar flow rates when inertial terms are negligible [53]. The linear relation between the pressure difference and velocity (see Equation (10)) means that any pressure value can be used as long as the numerical solver remains stable and the flow is dominantly laminar. Therefore, since the permeability of the catalytic surface is a characteristic of the layer and is independent of the volumetric flow rate of the medium, an exemplary pressure difference of 1 Pa was utilized. The set-up of the numerical laminar flow model is schematically presented in Figure 9a with symmetry flow condition defined on the upper and lower border of the geometry to introduce the flow continuity condition.

To produce a H${}_{2}$O${}_{2}$ concentration of 8% *v*/*v* out of an evaporation unit, 300 μL/s H${}_{2}$O${}_{2}$ solution 35% *w*/*w* is mixed with 2.5 m${}^{3}$/h air at 20 ${}^{\circ }$C and heated to an exit temperature of 270 ${}^{\circ }$C. The density of the H${}_{2}$O${}_{2}$ containing gas mixture is about 625 g/m${}^{3}$ with a dynamic viscosity of about 2.4 $\times 10^{-5}$ Pa·s. Figure 9b shows the numerical result of the velocity distribution using isothermal material properties and solving the laminar flow for a pressure difference of 1 Pa. Solving Equation (10) results in a permeability value of κ = 2.1 $\times 10^{-12}$ m${}^{2}$.

### 3.2. Decomposition of H${}_{2}$O${}_{2}$ with Porous MnO${}_{2}$ Catalyst

**Figure 10:**
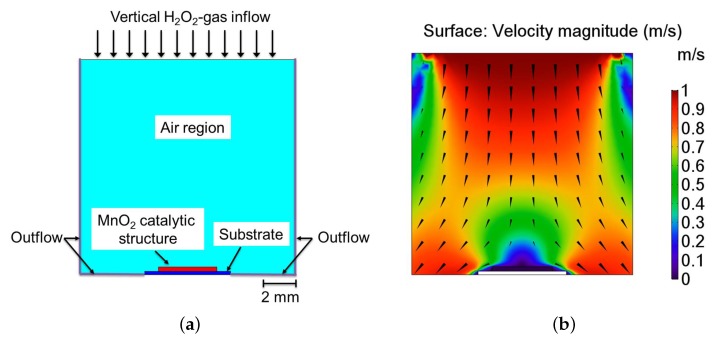
(**a**) Design of the numerical models representing a side view of the catalytic MnO${}_{2}$ structure (red) placed on top of a substrate (blue) with a vertical flow direction perpendicular to the sensor surface; (**b**) Result of the numerical model indicating the flow profile and direction.

In this section, the simulation results of the ϵ and κ values from the previous surface analysis of the catalyst are used to reflect the properties of the bulk porous catalyst. For this purpose, the ratio of pores (see Figure 6) in the bulk catalyst is assumed to be similar to the ratio of voids between MnO${}_{2}$ particles on the catalyst surface. In this context, a first hand approximation to analyze a H${}_{2}$O${}_{2}$ sensor structure subjected to a perpendicular flow of gaseous H${}_{2}$O${}_{2}$, as for conventional sensor measurement [28]. Thereby, the values of ϵ and κ through the catalyst structure are the previously derived values in the case of parallel flow direction (presented in Section 3.1). Hence, a homogenized two-dimensional (2D) model of the sensor is employed to analyze the H${}_{2}$O${}_{2}$ decomposition reaction. Figure 10a overviews the design of the homogenized 2D model that is used to analyze the sensor position relative to a flow of H${}_{2}$O${}_{2}$ containing gas. Due to the undetermined flow condition during sensor operation, an exemplary flow of 1 m/s at 270 ${}^{\circ }$C was presumed perpendicular to the surface of the catalyst.

Figure 10b shows the numerical result of the flow simulation. The perpendicular flow over the catalyst surface induces a pressure region that causes a decrease of velocity above the catalyst and the formation of a stagnation region above the sensor surface. The decrease of velocity impedes the mass transport of H${}_{2}$O${}_{2}$. Nevertheless, mass transport to the catalyst is not only governed by a convective turbulent flow, but also by the diffusion of material as defined by the steady-state Fick’s first law of diffusion given in Equation (11).

(11) ∇·(−D∇c)+u·∇c=−kc

In Equation (11), *D* is the binary diffusion coefficient of H${}_{2}$O${}_{2}$, determined for the current H${}_{2}$O${}_{2}$ containing gas by applying the Chapman-Enskog theory; for justification and detailed derivation we refer to ref. [28]. The variable u represents the velocity in m/s, *c* is the concentration of H${}_{2}$O${}_{2}$ in the flow in mol/m${}^{3}$, and *k* is the reaction rate in 1/s. At a H${}_{2}$O${}_{2}$ temperature of 270 ${}^{\circ }$C, the value of *D* is $4.19\times 10^{-5}$ m${}^{2}$/s.

**Figure 11:**
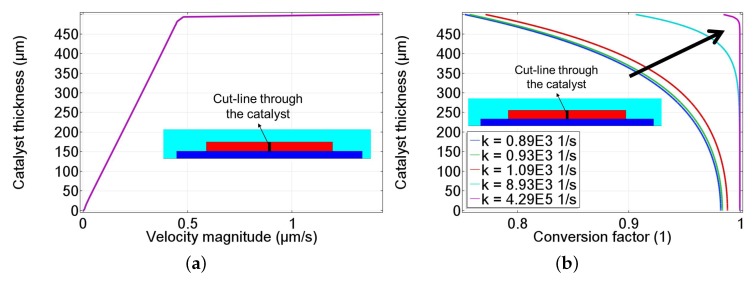
Plots of the numerical model through the center of the 500 μm MnO${}_{2}$ catalyst showing (**a**) the velocity magnitude in μm/s and (**b**) the conversion factor of H${}_{2}$O${}_{2}$ due to the catalytic decomposition reaction through the catalyst by varying the reaction rate (*k*) that increase in the direction of black arrow.

Figure 11a depicts the velocity profile across the mid of the catalyst (refered to as cut-line). Due to the porosity and permeability of the porous catalyst, the flow decreases through the catalyst reaching a value of 0 m/s at the substrate surface. Additionally, due to the uncertainty of the Arrhenius pre-exponential parameter ($A_{0}$) indicated in Table 2, a parametric sweep of the value of *k* is analyzed. Figure 11b shows the conversion factor of H${}_{2}$O${}_{2}$ through the catalyst center (cut-line). The conversion factor describes the amount of reacted H${}_{2}$O${}_{2}$ through the catalyst. A conversion factor of 1 represents the depletion of all H${}_{2}$O${}_{2}$ in the catalyst. It is observed that with an increase in the value of *k* (in the direction of the black arrow), the reaction occurs dominantly on the surface of the catalyst. This indicates a dominant surface decomposition reaction as observed in previous modeling attempts of the full three-dimensional (3D) sensor model described in ref. [28].

## 4. Conclusions

A short review on the catalytic decomposition reaction of H${}_{2}$O${}_{2}$ on MnO${}_{2}$ catalyst was presented. The differential design of a calorimetric H${}_{2}$O${}_{2}$ gas sensor was introduced and the thermocatalytic sensor principle was explained by an exemplary sensor response curve. The MnO${}_{2}$ catalyst was studied by SEM and XPS. XPS analyses indicate a change of oxidation state of the MnO${}_{2}$ catalyst which is on par with the decomposition scheme of H${}_{2}$O${}_{2}$. On the one hand, SEM analyses show a change in the roughness of the MnO${}_{2}$ catalyst surface after conditioning with H${}_{2}$O${}_{2}$ gas. On the other hand, XPS analyses result in a difference in the strength of the recorded signals before and after the conditioning process. The XPS analyses confirm the made observation from SEM analyses that the SU-8 overlayer is removed by H${}_{2}$O${}_{2}$ exposure. A numerical model that solves Darcy’s law on a geometry built from SEM analyses was used to derive physical parameters of the MnO${}_{2}$ catalyst, which include the surface porosity and permeability to H${}_{2}$O${}_{2}$ containing gas. Later, a homogenized 2D model was designed to study the effect of the MnO${}_{2}$ catalyst’s reaction rate on the rate of H${}_{2}$O${}_{2}$ conversion in the catalyst material. In a future extension of this work, combining the presented analysis method and three-dimensional sensor modeling described in ref. [28], a more precise model for a calorimetric H${}_{2}$O${}_{2}$ sensor can be achieved.

## Figures and Tables

**Table 1:** Specifications of hydrogen peroxide solution for indirect use in food processing.

Parameter	Specification
Phosphate	≤0.005% *w*/*w*
Iron	≤0.5 mg/kg
Tin	≤10 mg/kg
Lead	≤4 mg/kg
Residue on evaporation	≤0.006% *w*/*w*

**Table 2:** Summary of reaction kinetics as reported in literature and the derived reaction rate (*k*) at a gas temperature of 270 ${}^{\circ }$C (543.15 K).

$A_{0}$, 1/s	$E_{a}$, kJ/mol	*k* (Derived for 543.15 K), 1/s	References
8 $\times 10^{10}$	54.8	4.29 $\times 10^{5}$	[45]
1 $\times 10^{8}$	52.5	$0.89\times 10^{3}$	[46]
1 $\times 10^{9}$	52.5	$8.93\times 10^{3}$	[47,48]
6–7 $\times 10^{7}$	50	0.93–1.09 $\times 10^{3}$	[43]
